# The misuse of colour in science communication

**DOI:** 10.1038/s41467-020-19160-7

**Published:** 2020-10-28

**Authors:** Fabio Crameri, Grace E. Shephard, Philip J. Heron

**Affiliations:** 1 Centre for Earth Evolution and Dynamics (CEED), University of Oslo, Postbox 1028, Blindern, 0315 Oslo, Norway; 2 Department of Earth Sciences, Durham University, Durham, UK

**Keywords:** Software, Scientific community

## Abstract

The accurate representation of data is essential in science communication. However, colour maps that visually distort data through uneven colour gradients or are unreadable to those with colour-vision deficiency remain prevalent in science. These include, but are not limited to, rainbow-like and red–green colour maps. Here, we present a simple guide for the scientific use of colour. We show how scientifically derived colour maps report true data variations, reduce complexity, and are accessible for people with colour-vision deficiencies. We highlight ways for the scientific community to identify and prevent the misuse of colour in science, and call for a proactive step away from colour misuse among the community, publishers, and the press.

## Abstract (web summary)

The accurate representation of data is essential in science communication, however, colour maps that visually distort data through uneven colour gradients or are unreadable to those with colour vision deficiency remain prevalent. Here, the authors present a simple guide for the scientific use of colour and highlight ways for the scientific community to identify and prevent the misuse of colour in science.

## Introduction

Vision is one of the most fundamental means of communication. It is (or should be) in every scientist’s best intention to make figures and their content as accurate and easily understandable as possible. One of the most powerful aspects of images is colour, which in turn transforms information into meaning. The visual evaluation of a colour gradient is important to a variety of different fields such as the first direct impression of a black hole^1^, the mapping of votes cast in political elections^2,3^, the planning of an expensive rover route on Martian topography^4^, the essential communication of climate change^5,6^, or the critical diagnosis of heart disease^7^. However, when colours are used incorrectly, this can lead to the effective manipulation of data (e.g., by highlighting some data over others), the oversight of the needs of those with colour vision deficiencies, and the removal of meaning when printed in black and white (Supplementary Note 1).

As science has become more prevalent in mainstream culture, it is not only the scientific community that suffers due to the use of poor colour choices, but also the wider public. Colour maps, therefore, are a crucial intersection between science and society. For instance, weather forecasts and hazard maps are two examples of immediately societal-relevant data sets that are also repeat offenders for use of the rainbow-like colour maps. Given the (daily) importance of these scientific topics, the underlying data should be conveyed in a universal manner. However, the colour-vision deficient fraction of the population is excluded and therefore unable to process this critical information. Furthermore, zones of danger, such as the boundaries of a hurricane track or current virus spread, are often based on uneven colour gradients to accentuate their importance. Using an uneven colour gradient is not an action without consequences, including those with significant financial or life-threatening consequences. Decisions based on data being ‘unfairly’ represented could produce, for instance, a Martian rover being sent over terrain that is too steep as the topography was inaccurately visualised, or a medical worker making an incomplete or inaccurate diagnosis based on uneven colour gradients.

Although some scientific communities have largely moved away from using distorting colour maps, such as rainbow, there are numerous signs of bad habits returning en masse^8^. Unfortunately, the previous efforts within specific disciplines to discredit rainbow-like maps appear to have not trickled through to all scientific leaders, publishers, and software designers. For most scientists, the choice of colour maps has become almost passive, with the unscientific rainbow-like colour palette being commonplace. Similarly, colour maps that pair red–green are also problematic, but remain widely used. At one point, most of the common software programmes applied rainbow as their default palette (e.g., MatLab, Paraview, VisAd, IrisExplorer) despite issues surrounding colour maps like rainbow, and variations thereof, being known for some time^9–20^.

**Fig. 1:**
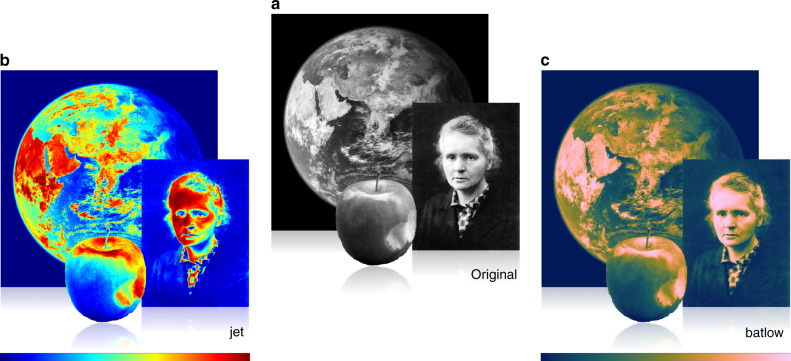
The superiority of scientifically derived colour maps. By knowing what something looks like in advance, the distortion by unscientific colour maps, like jet or rainbow, becomes instantly obvious. The look of scientific data is, however, usually unknown a priori, which makes the distortion of an unscientific colour map, and the true data representation of a scientifically derived colour map, like batlow^41^, less apparent. Marie Skłodowska-Curie, as originally photographed by Henri Manuel around 1920, the Earth from space, and an apple are shown **a** in their original images and **b** in distorted and **c** in undistorted colour versions. Inferring the true picture from an unscientifically (e.g., jet) coloured data set is incomparably harder than from a data set represented in a perceptually uniform and ordered colour map, like batlow^41^.

**Fig. 2:**
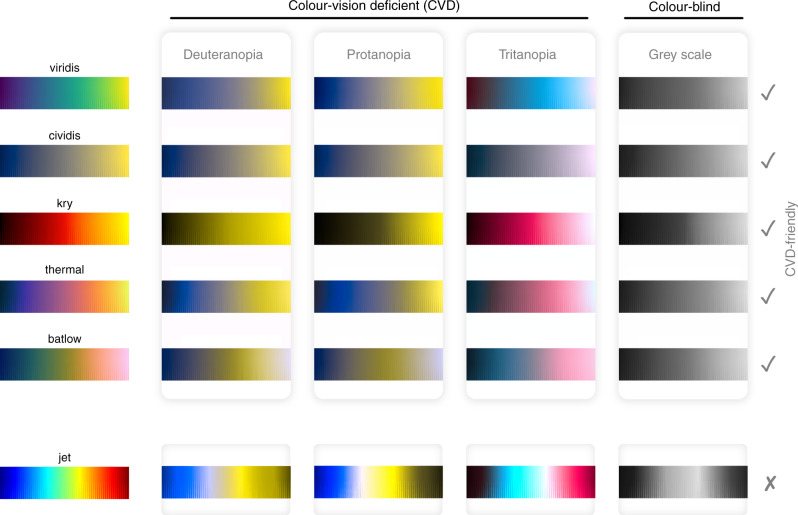
Colour vision tests. Available perceptually uniform colour maps versus the non-uniform rainbow (i.e., jet; bottom row) as seen with either of the three common forms of human colour-vision deficiency (deuteranopia, protanopia, and tritanopia), and for grey-scale (representing total colour-blindness or simple black-and-white prints). Rainbow, the most-widely used colour map, fails to reproduce a meaningful smooth gradient, yet the other colour maps (see Box 2) are all universally readable.

So, what’s the problem with these colour maps? Even though rainbow colour maps might reflect aesthetic attractiveness, the extreme values in the standard Red-Green-Blue (RGB) are very dominant and can, therefore, distract from the underlying visual message^21^. In rainbow colour maps, the yellow is the brightest colour and attracts the eye the most^22,23^ (see Box 1), but it is neither at the end nor the centre of the colour map, while its greenish shades form a wide band with low perceived colour contrast (Supplementary Fig. 1). Hence, such an arrangement of colours can unfairly highlight a particular section of the parameter space while obscuring other parts (Fig. 1). Building a colour map on a purely physical rather than a perceptual basis significantly alters how we perceive data; it adds artificial boundaries to some parts of the data range, hiding small-scale variations elsewhere, it prevents any visual intuitive order occurring in the data set, and renders the data unreadable for readers with common colour-vision deficiencies (Fig. 2).

Colour maps that include both red and green colours with similar lightness cannot be read by a large fraction of the readership (Fig. 2). The general estimate is that worldwide 0.5% of women and 8% of men are subject to a colour-vision deficiency (CVD; e.g., refs. ^24,25^, and references therein). While these numbers are lower and almost disappear in populations from sub-Saharan Africa, they are likely significantly higher in populations with a larger fraction of white (Caucasian) people as, for example, in Scandinavia^26^. It is needless to state that scientific results should be accessible to as many people as possible. CVD-friendliness is, therefore, an aspect that should be important to all researchers, as well as publishers^18,27,28^.

For the casual reader, it might appear curious that the community of scientists, a group of people who are usually more critically inclined, fail to condemn the proliferation among themselves of rainbow and other, similarly unfit, colour maps. Based on this prevalence of unscientific colour maps, a basic understanding of their nature (Supplementary Fig. 2) and their dynamics seems to be missing in the academic community. Colour map choice is often made passively and is not subject to the same scrutiny as other data methodologies. Rainbow’s eye-grabbing nature, ease of use, and the inertia of both software products and scientific group leaders, allows the scientific community to remain attached to this unscientific colour map^29^. As a result, pointing out colour map flaws to the science community through scientific peer-review remains a challenging task, as requests or suggestions to change the colour palette are often met with rebuttal, misunderstanding, or are simply ignored.

Here, we highlight the importance of the scientific use of colour in data visualisation. We summarise methods to generate colour maps that deliver equal colour gradients all along the colour axis, to prevent data distortion. Various types of scientifically derived colour maps are readily and freely available (Box 2), and are the only way to intuitively and inclusively represent data without any blind interpretation. We outline key guidelines for choosing scientific colour maps that most accurately represent your data, and highlight how to spot common unscientific colour maps so they can be avoided. Finally, we call for a universal switch by the science community to adopt scientifically derived colour maps. Individuals, publishers and press should take a proactive approach to spot unscientific use of colour and so prevent the dissemination of visually distorted data.

BOX 1: Human colour perception Colour is not inherent in objects. A perceived colour is the portion of light that is reflected from a surface and translated into a specific colour by our eyes and brain (Box Fig. 1). A certain colour is perceived via its hue, the true tint (i.e., yellow, orange, red, violet, blue or green), and its luminosity (the measure of how bright or dark a hue is). Within the eye, one type of light receptors, the rod cells, process achromatic information (i.e., lightness or grey-scale vision), which is derived mostly from the light’s energy. The other type, the cone cells, handle chromatic information (the hue) mostly from the light’s wavelength. The latter transmit information to the brain and create the sensation of colour that is so familiar to most of us.

Two thirds of all cone cells process longer wavelengths of light (i.e., colours like red, orange, yellow), which allows the human eye to perceive more colour detail across warmer colours than for cooler colours^54^. Greenish colour gradients tend to under-represent a given data variation compared to yellow-red gradients. Three types of cone cells (for short, medium and, long wavelengths) build the trichromatic visual system that can, as a whole, represent all colours in our visual spectrum^55–58^.

The physiological prerequisites for perceiving colour suggest that there is no uniform colour perception among individuals^59^; several physiological deviations can lead to a shift in colour perception, which, in general, is hardly measurable^60^. However, it is not unlikely that at least one of the cone cell types is altered, defect, or even absent, which induces a significant shift in colour vision. Such a shift is commonly referred to as colour-vision deficiency (CVD), or colour blindness, and can be modelled by a given combination of the three fundamental spectral sensitivity functions representing short, medium and long wavelengths of the light^55^ (as represented in Fig. 2). The most common form of colour-vision deficiency is the red–green dichromatism, called deuteranomaly, concerning the M-cone and causing red and green to appear indistinguishable. Protanomaly, concerning the L-cone, and tritanomaly, concerning the S-cone cause reduced sensitivity to red and blue light, respectively. Total colour blindness is, fortunately, very rare, but does exist.

Box Fig. 1: **Simplified schematic of human colour perception**. The perception of a certain colour, or colour gradient, by the optical cortex depends not only on the optical properties of the object, but also on the optical properties of both light source and object background, and the eye’s light receptors (rod and cone cells for short, medium and long wavelengths).
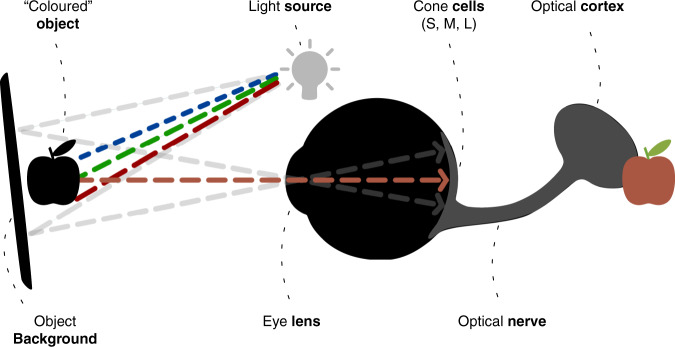

BOX 2 Freely available scientifically derived colour maps and toolkits Scientifically derived colour maps should be easily recognisable, and also be freely and readily available. While there are many online sources to download pre-made colour maps, and toolboxes to create colour maps, only few offer all the aspects required for use in science communication. Below, we mention the most accessible and/or robustly documented sources of scientifically derived colour maps, and outline their individual properties.

- **Colorbrewer**: The Colorbrewer colour maps (available at ref. ^61^) developed by Cynthia Brewer are provided through an online tool to manually produce and export a variety of discrete colour maps, which can, optionally, be colour-vision deficiency friendly and exported to a given a variety of formats. With the main aim being cartography, the online tool offers sequential, diverging and categorical (or in other words, qualitative) colour maps, but does not currently offer them in a continuous type.
- **MPL** (Matplotlib): The MPL colour maps (available at ref. ^62^) developed by Stéfan van der Walt and Nathaniel Smith. MPL maps aim for the most accurate perceptual uniformity with its widely applied colour maps being: viridis, magma, plasma and inferno. These maps have spearheaded the way towards more scientific colour mapping. The MPL colour maps are all sequential and continuous only. The MPL colour maps are openly available (currently for use with Python) and often built into software.
- **Cividis**: The cividis colour map^38^ (available at ref. ^63^), developed by Jamie R. Nuñez and colleagues, aims to represent an almost identical appearance for red–green colour-vision deficiencies, the closest of all currently available colour maps, while also being perceptually uniform. The colour-vision deficiency friendly, sequential and continuous colour map is currently available as a standard colour array.
- **CMOcean** (colormaps inspired by oceanography): The CMOcean colour maps^20^ (available at ref. ^64^), developed by Kristen M. Thyng and colleagues, aim to provide the most intuitive colours for a given suite of physical parameters, while now also being perceptually uniform. A variety of continuous sequential, diverging and cyclic colour maps are provided to allow for an intuitive, true representation of a given physical parameter field. The CMOcean colour maps are available in a large variety of file formats.
- **CET** (Centre for Exploration Targeting): The CET colour maps^32^ (available at ref. ^65^), developed by Peter Kovesi, aim to offer a large choice of the most common colour combinations in a wide variety of data formats. Many of the offered colour maps feature perceptual uniformity, although not all of them to the highest standards. The CET colour maps are continuous and cover sequential, diverging, and cyclic classes.
- **Scientific colour maps**: The Scientific colour maps^30^ (available at ref. ^41^, and permanently archived at ref. ^52^) are perceptually uniform (based on the underlying methodology of the CET colour maps^65^), perceptually ordered, colour-vision deficiency and colour-blind friendly, readable in black and white prints, and, if applied properly, also data set specific and parameter intuitive. The Scientific colour maps include sequential, diverging, and cyclic palettes, which are also provided as discrete and categorical palettes, and are available in a multitude of different file formats. They are also available through external routines and as built-in versions in a variety of software programmes. In contrast to others, the Scientific colour map package includes individual colour map diagnostics, and is versioned on a long-term online repository so individual versions can be accurately cited, which allows active developments from the community (e.g., improve their perceptual uniformity to the latest standards), and aids overall scientific reproducibility.

### The importance of colour to prevent data distortion

**Fig. 3:**
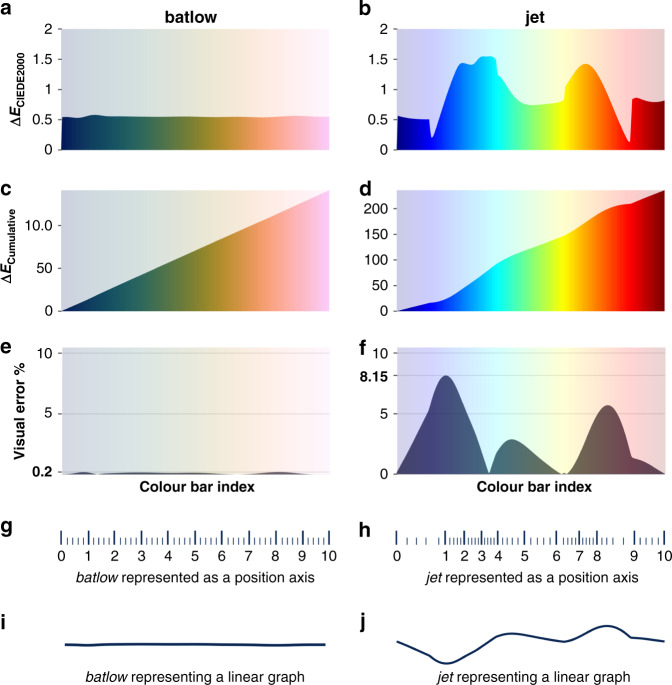
Colour map measure, distortion, and error. **a**, **b** The incremental lightness difference, Δ*E*, here using the *CIEDE*2000 formulation (see ‘Methods’), is a measure for the perceptual colour difference along the colour map. For a perceptually uniform colour map, Δ*E*_*CIEDE*2000_ should be equal all along the colour map (i.e., a flat graph; **a**). Using **c**, **d** the cumulative colour lightness difference, Δ*E*_Cumulative_, it is possible to extract **e**, **f** the resulting visual error in percentage of total data variation. For scientifically derived colour maps like batlow^41^, the resulting error introduced to the data by the colouring is negligibly small as **g** the incremental data variation is represented equally all along the axis, and a linear data gradient, therefore, appears linear. Put differently, **i** a flat line looks flat. For non-scientific colour maps, like jet, **h** data gradients are unevenly represented and **f** visual error can be >7% of the displayed data variation such that **j** a linear graph (e.g., a flat line), for example, becomes unrecognisable.

Scientific data should be displayed without the addition of visually artificial features or subtraction of real details, while also being universally readable and intuitive. A perceptually uniform colour map weights the same data variation equally all across the dataspace, while other colour maps (such as rainbow) interpret some small data variation to be more important than others (Fig. 3). For such unscientific, perceptually non-uniform colour maps (like rainbow), the interpretation is performed blindly (i.e., by the colour map instead of by the author) as the author or reader is not aware of the exact visual distortion that is introduced by the colour map (Fig. 3). Any scientific study drawing conclusions from a given set of data is, therefore, strongly dependent on the perceptual uniformity of the colour palette applied and, with it, author and reader become captive to a blind interpretation (for various examples, see Supplementary Note 1). Such a colour-introduced blind interpretation can diverge from an objective representation by more than seven percent of the displayed data variation^30^ (Fig. 3e–f). A flat slope on Mars can become visually distorted by the most prominent local gradients in colour rather than data, and then appears like rough terrain, while rough terrain conversely might be interpreted as a flat slope (compare Supplementary Fig. 7a, b), which is suboptimal with regards to a Martian exploration.

Scientifically derived colour maps are perceptually uniform. This means the same data variation is weighted equally across the dataspace, and so the true data variation is accurately represented without unnecessary visual error (Fig. 3a, c, e, g, i). Just like a spatial *x*-, *y*-, or *z*-axis needs to have equal spacing between all axis tick marks (Fig. 3g, h), a colour axis (commonly termed colour bar; Supplementary Fig. 1) needs to have equidistant colour gradients. In other words, a certain data variation (e.g., a 5 °C temperature drop) should appear the same no matter whether it occurs at low temperatures (–10 °C) or high temperatures (30 °C).

### Design and implementation of scientifically derived colour maps

While understanding the importance of perceptually uniform colour maps is simple, creating them can be complicated. Quantifying perceptual colour gradients is challenging because the complex nature of the human eye and brain has to be considered (Box 1). However, extensive research has been conducted to understand and provide guidelines for an optimal colour map design (see description in ‘Methods’ and refs. ^18–20,29,31–35^), and today, various freely available tools exist to create perceptually uniform colour palettes. In Box 2, we have compiled freely available scientific colour map toolkits, and described the benefits and limitations of each toolkit, to aid users in designing and/or selecting the most appropriate and accurate colour map for their data.

**Fig. 4:**
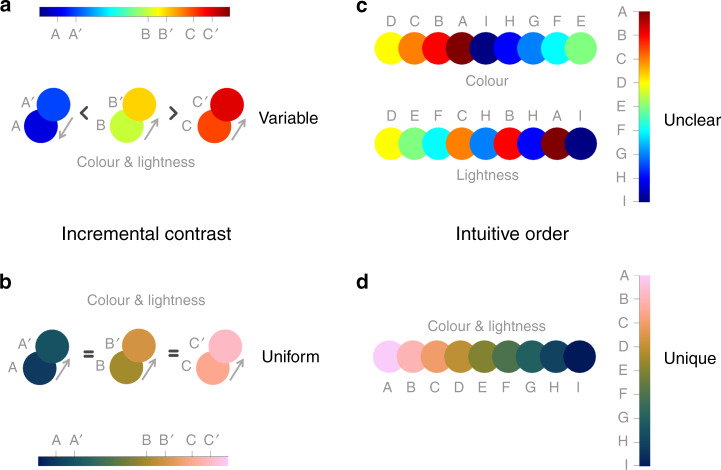
Perceptual uniformity and order. A constant incremental colour and lightness contrast along a colour map is a proxy for its perceptual uniformity. **a** While a certain incremental data variation is either under- or strongly overrepresented with jet (a.k.a. rainbow) depending on the colour map segment, it is instead **b** evenly represented all along a colour bar when using a scientific colour map like batlow^41^, due to its uniform colour and lightness contrast. Perceptual order is given when individual colours of a colour map can be sequentially ordered effortlessly without consulting the colour bar. While **c** a sequential ordering is not intuitively possible for jet (a.k.a. rainbow), it is **d** possible to sequentially order individual colours of a scientifically derived colour map like batlow^41^, thanks to its constant lightness gradient.

Perceptual uniformity is a crucial property for colour maps used in science, but by no means the only property to care about. Perceptual colour order is another important aspect in scientific colour map design, as it ensures the colour gradient is easily and intuitively understandable and allows qualitative understanding of a data set (Fig. 4). To achieve perceptual order, both lightness and brightness should increase linearly to avoid the perception of artificial gradients and to easily discern and compare significant values. The heated black-body radiation palette, for which the colours can be easily ordered from black-red-orange-yellow (or vice versa), is one example. Perceptual order is, therefore, a great asset to a colour map by emphasising gradients and pattern(s) in data. Moreover yet, colour maps specifically created for certain data sets (e.g., ref. ^36^) by no means guarantee their perceptual uniformity. Perceptual uniformity and intuitive colour order are both needed to prevent bias percolating into colour maps. This is also the case for so-called ‘improved’ rainbow-like maps such as Google’s Turbo^37^. Although Turbo appears to meet perceptual order, the perceptual uniformity requirement of a science-ready colour map is not met due to its non-uniform lightness spectra.

In addition, colour maps should be universally readable, and they can be mathematically optimised to account for colour-vision deficiency using modern colour appearance models^38^ (Fig. 2 and Box 1). As well as making colour palettes readable for readers with variable vision capabilities, it is also advantageous to make them readable for completely colour-blind readers. In contrast to other colour palettes, a colour map for scientific use should feature a uniform gradient across the whole colour axis. With an even, monotonic lightness gradient (Supplementary Fig. 1a), a colour palette remains readable (as well as perceptually uniform and ordered) even after a conversion to grey scale, assuming no complicated grey-scale filter is used.

**Fig. 5:**
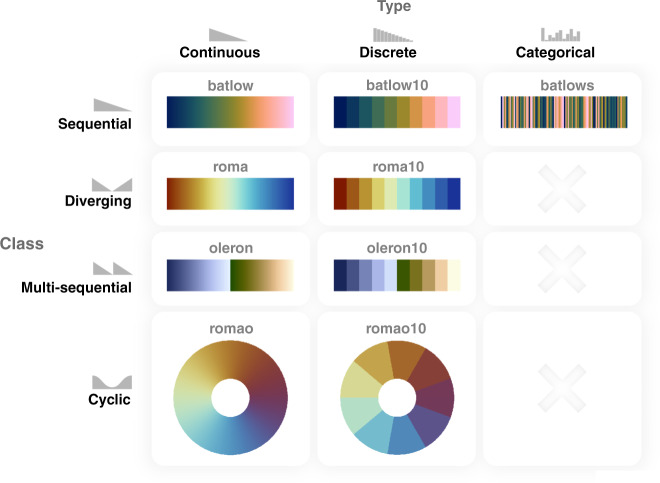
Colour map classes and types. The various classes of colour maps (*sequential*; *diverging*; *multi-sequential*; *cyclic*) and types (*continuous*; *discrete*; *categorical*). Only sequential colour maps can be faithfully applied to categorical types of data.

**Fig. 6:**
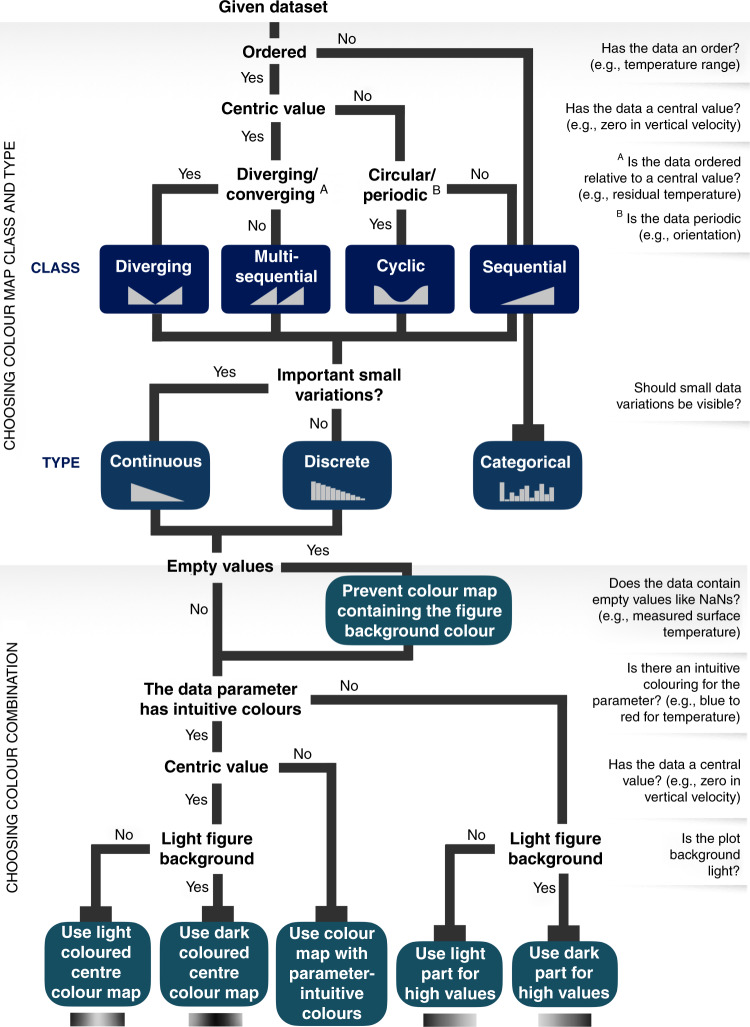
Guideline for choosing the right scientific colour map. For effective data representation, the nature of a given data set has to be matched by a suitable colour map class, type, and colour combination.

To achieve the best possible data representation, colour palettes need to effectively convey the underlying data and its nature. This can be achieved by choosing the most appropriate colour map class and type (Fig. 5 and outlined in detail in Supplementary Notes 2 and 3). If the data is divergent about a central value (e.g., centred about zero), a divergent or a multi-sequential colour map should be chosen that clearly and intuitively distinguishes either side of the axis (Fig. 5, and Fig. 6 for a detailed user guide).

An intuitive colour gradient becomes imperative if data are displayed without the provision of the colour bar (the axis relating colours to data values). Unfortunately, missing colour bars are more common than most would expect, as scales are sometimes cropped out or omitted during reproduction or subsequent dissemination (e.g., refs. ^1,4,39^). It is imperative to ensure the colour bar is included on all figures where a colour scale is used, as even the most intuitive colour map could be rendered useless with no colour bar for the reader to refer to. Excluding a colour bar would be equivalent to not including the axis labels and tick marks on the *x*- or *y*-axis of a plot. Additionally, attention has to be paid to the lightness (i.e., light or dark) of the background on which the colours are displayed. Background creates contrast, in lightness and colour, to the displayed data (Supplementary Fig. 3 and Supplementary Movie 1).

To optimise the use of the currently available perceptually uniform colour maps listed in Box 2, there are certain requirements to prevent them from becoming distorted (e.g., ref. ^30^). As the colour bar has to be handled similarly to the position axis, parts of a scientifically derived colour map cannot be subsequently deformed by being partly squeezed or elongated. If the spacing between a position axis’ ticks were squeezed or elongated, it would visually distort the data in the figure. Simply put, a linear graph would no longer appear linear (Fig. 3h, j). Therefore, altering any available scientifically derived colour map is not recommended. Additionally, avoiding certain types of graphs and plots is important to not alter the local perception of individual colours. Common heatmaps with directly connected colour tiles, as opposed to tiles with gaps in between, can alter individual colours significantly^40^ (Supplementary Fig. 3).

### How to recognise an unscientific colour map

It is the responsibility of individuals, publishers, and the press to prevent the dissemination of visually distorted data and to take a proactive approach in spotting the unscientific use of colour. There are straightforward checks to recognise the unscientific use of colour that are handy for users, readers, reviewers and editors:

1. The colour bar should be perceptually uniform to prevent data distortion and visual error. This means the perceptual colour differences between all neighbouring colours should appear the same. If two neighbouring colours have a different variation compared to other neighbouring colours (e.g., two greenish versus two yellowish-reddish colours in rainbow colour maps), the colour bar is perceptually non-uniform and not scientific (as shown in Fig. 4).
2. The colour map should not contain red and green at a similar luminosity (Box 1 for definitions). If this is the case, it can be assumed that these two colours cannot be distinguished by a large fraction of the readership and, therefore, fails as a scientific way of displaying data.
3. The common rainbow colour map should not be used in data visualisation. There is not a single rainbow colour map with similarly bright colours across the colour bar that comes close to being scientific (e.g., ref. ^32^).
4. The most secure test is to discard any colour map that is not described as scientifically derived (for example, is not listed in Box 2), as it should be both perceptually uniform and CVD friendly.

### A proactive step forward for the science community

It is important to maintain the progress that has already been made across the scientific community (in particular in the fields of Earth, Space, and Climate science). Recently, a number of significant scientific achievements have been based upon non-distorting and universally readable scientifically derived colour maps, such as the first ever observation-based visualisation of a black hole^1^, for which a lajolla-like colour map^41^ was used, and the effective visualisation of climate change on a local, regional, and global level^39^, which applied a vik-like colour map^41^. However, there are danger signs that this progress could be lost. Unscientific colour maps are still often set as the default in software packages, which in turn also renders them a prevalent choice among many academic group leaders. Furthermore, unscientific colour maps are still accepted and published by academic journals. This combination leads users, students, and readers into believing that these choices are based on informed decisions, and that there are no fundamental issues with unscientific colour maps. Given this current landscape, it is an instructive reminder that new generations of scientists have to be made aware of the importance of scientifically derived colour maps and the pitfalls of those that are not.

Transferring the knowledge and awareness about the importance of scientifically derived colour maps to new generations of scientists is, therefore, a key goal. Teaching is a frontline tool in building solid visualisation skills. Indeed, learning and applying scientific data visualisation should be a requirement to receive BSc, MSc, and PhD degrees. Here, we provide an instructive user guide for choosing and applying a suitable scientific colour map (Fig. 6), which should be part of every research office, and possibly even desk space. We provide a poster (Supplementary Data 1) that can be placed in communal areas, like near a printing station, coffee machine or restroom, that highlights the key advantages of scientifically derived colour maps and serves as a conversation starter.

Recommendations to colleagues via peer-review is another critical tool to ensure the quality of our ongoing scientific visual communication. While editors could certainly provide guiding recommendations, it is also critical that scientifically derived colour maps are being applied already during the early diagnosis of the data, and not just applied for publication only. Set instructions by scientific journals or conference organisers could remind researchers to fulfil the graphical standards that science needs to be built upon. It might even be useful to limit researchers to using only a handful of hand-picked, suitable colour maps for specific types of data and data parameters to streamline and enhance data visualisation. This type of approach is being developed for the next assessment reports of the Intergovernmental Panel on Climate Change (IPCC), in collaboration with Melissa Gomis (Graphics and Science Communication officer, IPCC WGI Technical Support Unit, Université Paris-Saclay, France).

Presently, scientifically derived colour maps are easily accessible and applicable across various tools and platforms and suitable palettes exist for any given data (Box 2). The open availability of such colour maps ultimately leaves little legitimate room to continue using colour maps that cause visual distortion, are unreadable in some circumstances, or exclude readers from understanding them.

The wider scientific community needs to accept that colour maps are a pivotal tool in scientific discovery, data visualisation and science communication. Non-scientific colour maps require scrutiny and rejection from the community to preserve academic integrity. The evidence is clear, there are no more reasons to continue using unscientific colour maps.

## Methods

### Defining colour spaces

Various methods and tools based on different metrics and colour spaces (Supplementary Note 4) exist to diagnose the uniformity of a colour palette (e.g., refs. ^32,34,38^). A widely used example is the International Commission on Illumination’s uniform colour space named CIELAB UCS^42–44^. CIELAB UCS (also abbreviated from Uniform/Unified Colour Scale, Chromaticity Scale, Chromaticity Space) describes the complete range of colours in a perceptually uniform rectangular coordinate system^45^ and allows the creation of perceptually uniform colour palettes^32,35,46,47^.

The colour appearance model CIECAM02^48,49^, and the perceptually uniform colour space CIECAM02-UCS^50^ based on it, is the current gold standard to describe how we perceive colour and colour differences. CIECAM02-UCS describes a certain colour by its lightness (L) and its red–green (a) and yellow-blue (b) correlates (Supplementary Figs. 4 and 5), and it represents a certain perceptual colour variation on equal Euclidean distances across the whole space. Colour spaces like CIECAM02-UCS make it possible to design perceptually uniform colour maps and, also, to take colour-deficient vision into account.

Here, the CIELAB UCS colour difference metric Δ*ECIEDE* 2000^51^ is used (Fig. 3a, b) and allows the calculation of a local lightness gradient between two colours by

1 $$\Delta {\mathrm{E}_{\it{CIEDE}2000}} = [(L_1 - L_2)^2 + (a_1 - a_2)^2 + (b_1 - b_2)^2]^{1/2},$$

where *L* is the lightness of one specific colour, and a its red–green and *b* its yellow-blue correlative (Supplementary Figs. 4 and 5). This lightness difference metric can be used to diagnose any colour map (e.g., ref. ^20^) and calculate the effective, perceptual error that is added to the underlying data by uneven colour gradients along a colour map^30^. These errors and the resulting visual data distortion can be significant and make, for example, linear data gradients look like a wobbly graph (Fig. 3).

## Supplementary information

Supplementary Information

Description of Additional Supplementary Files

Supplementary Data 1

Supplementary Movie 1

## Data Availability

The scientific colour maps are openly available from ref. ^41^ and archived on Zenodo^52^ (10.5281/zenodo.1243862). All other additional information related to the article is provided in the Supplementary Information and Box 2.
