# Supplementary material for: The misuse of colour in science communication
Source: Nat Commun. 2020 Oct 28;11:5444. doi: 10.1038/s41467-020-19160-7 (PMC7595127; doi:10.1038/s41467-020-19160-7)
Supplement: Supplementary file 1 — Supplementary Information [file 41467_2020_19160_MOESM1_ESM.pdf]

Supplementary Information for "The misuse of colour in science communication"

Fabio Crameri[1], Grace E. Shephard[1], Philip J. Heron[2]

[1] *Centre for Earth Evolution and Dynamics (CEED), University of Oslo, Postbox 1028 Blindern, 0315 Oslo, Norway*

[2] *Department of Earth Sciences, Durham University, Durham, United Kingdom*

Contents

Supplementary Figures **2**

Supplementary Note 1: Better figures of widely used example datasets **6**

Supplementary Note 2: Colour map classes **13**

Supplementary Note 3: Colour map types **14**

Supplementary Note 4: Colour spaces background **15**

Supplementary References **16**

Supplementary Figures

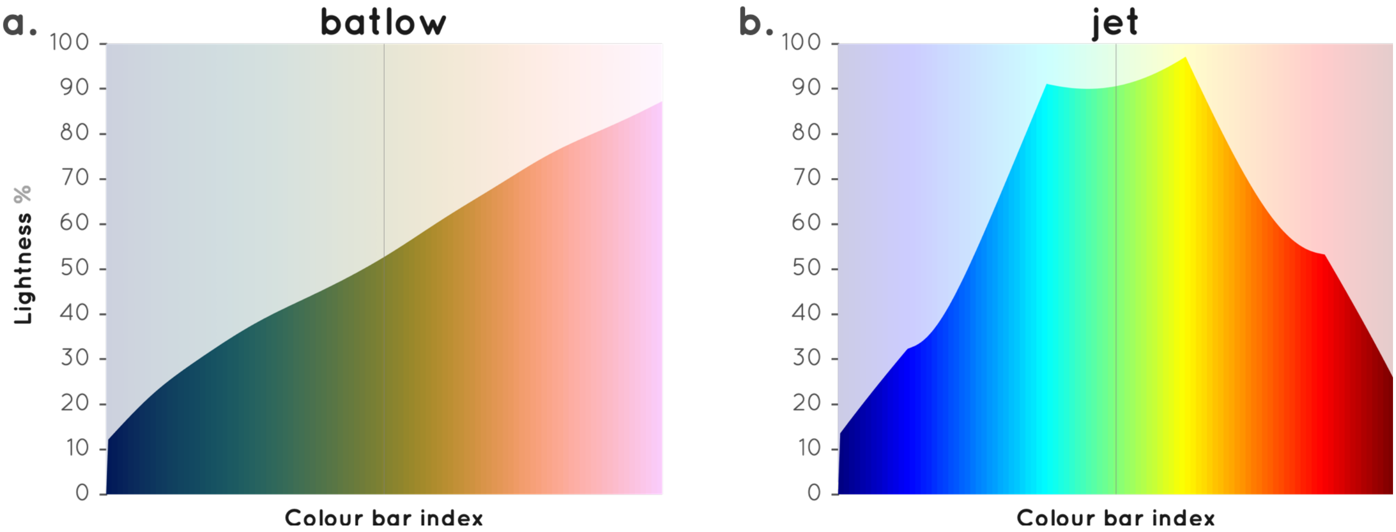

Supplementary Figure 1 Colour map lightness gradient. Lightness is a property of colour to which the human visual apparatus is most sensitive to and, as such, lightness is a pivotal property of a colour map. Shown are the lightness gradients across **a** *batlow*[1], for which it is monotonically increasing, and **b** *jet*, for which it is neither monotonically increasing, nor symmetric, nor has its maximum lightness in a sensible location.

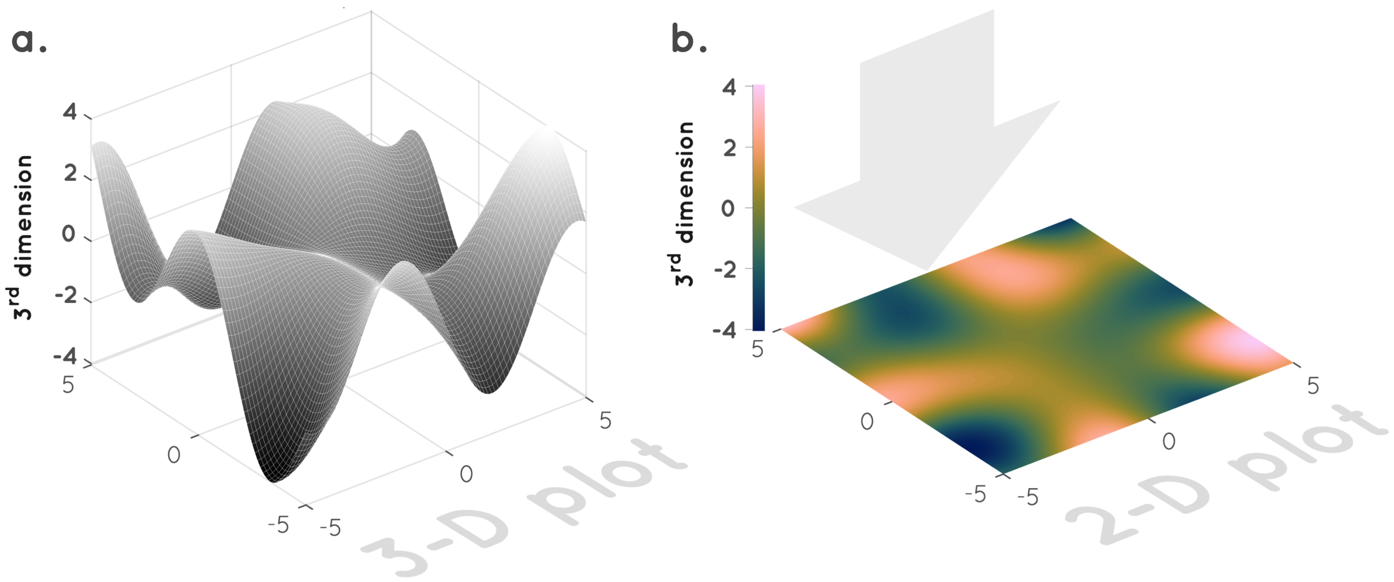

Supplementary Figure 2 The colour axis. Replacing a third position axis with a colour gradient (i.e., a colour axis) can conveniently display **a** a three-dimensional function in form of **b** a two-dimensional plot. The data variation across the 3rd dimension is only accurately represented if **a** the distance (i.e., position variation) and **b** the colour variation across a certain data range (i.e., colour bar) are constant.

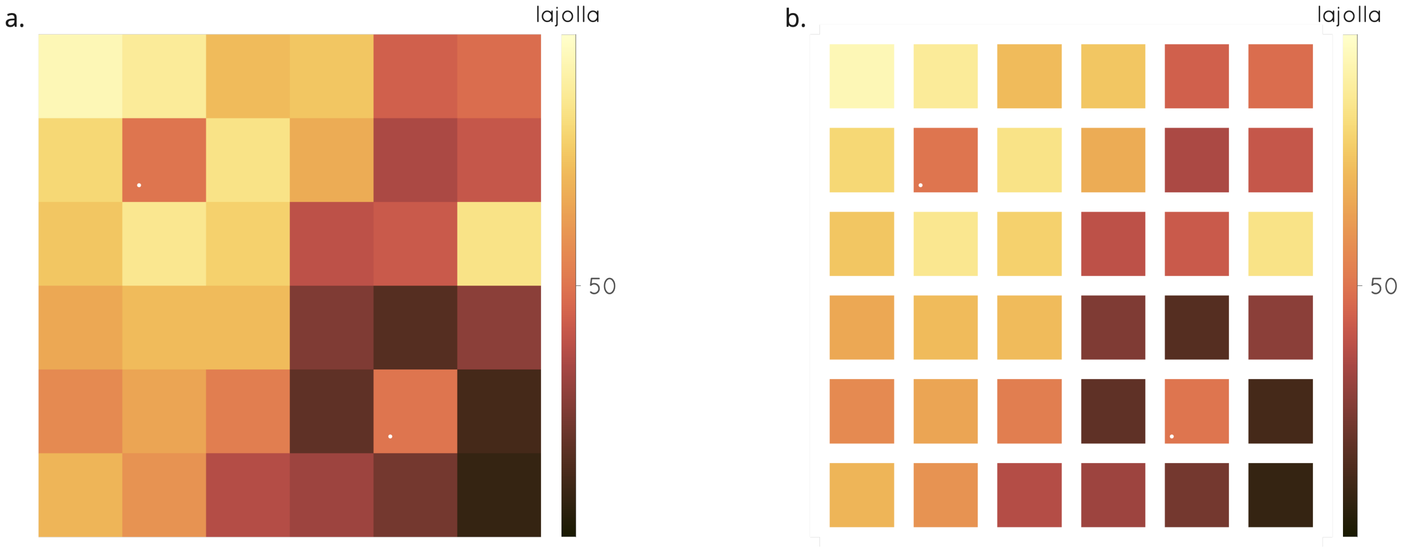

Supplementary Figure 3 The importance of background colouring. **a** The two tiles marked with a white dot misleadingly appear to have slightly different colours even though they do not and indeed represent the exact same value (i.e., 50) as indicated on the colour bar featuring the Scientific colour map *lajolla*[2] (an effect also known as "checker shadow illusion"). **b** A common suggestion to prevent such background-induced perceptual distortion for such so-called 'heatmap' plots is to add an equally coloured space surrounding the individual tiles and separating them from each other. However this seems to be a marginal improvement only and superposing the actual numbers on the tiles seems necessary.

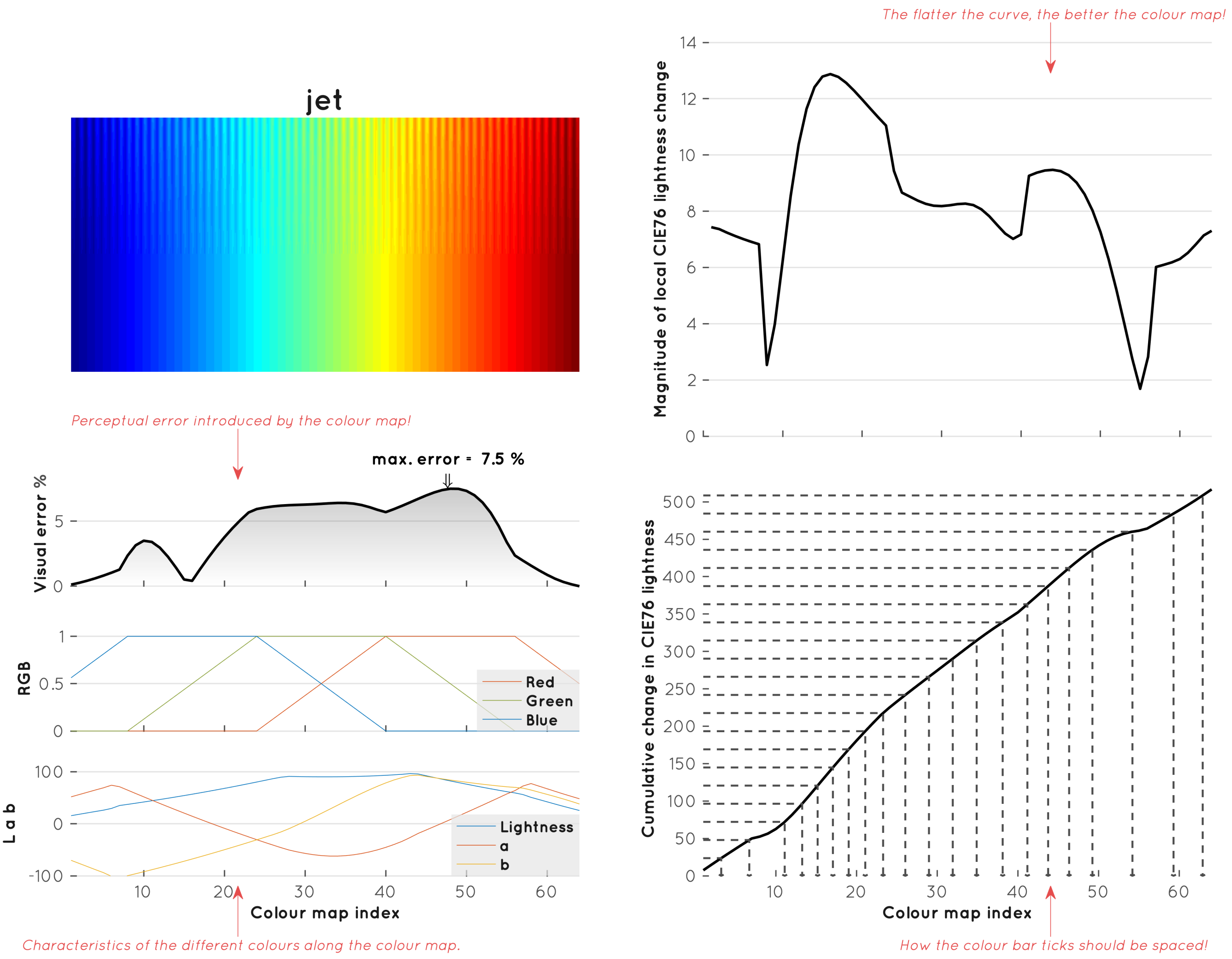

Supplementary Figure 4 Colour map diagnostics for jet (a.k.a. rainbow). The figure shows the colour map with low gradient ripples to indicate how it represents low-contrast regions in different locations of the colour bar, and a plot of the lightness difference, ΔE, indicating better perceptual-uniformity the flatter the curve. Diagnostics are performed after Kovesi [3] and Crameri [4].

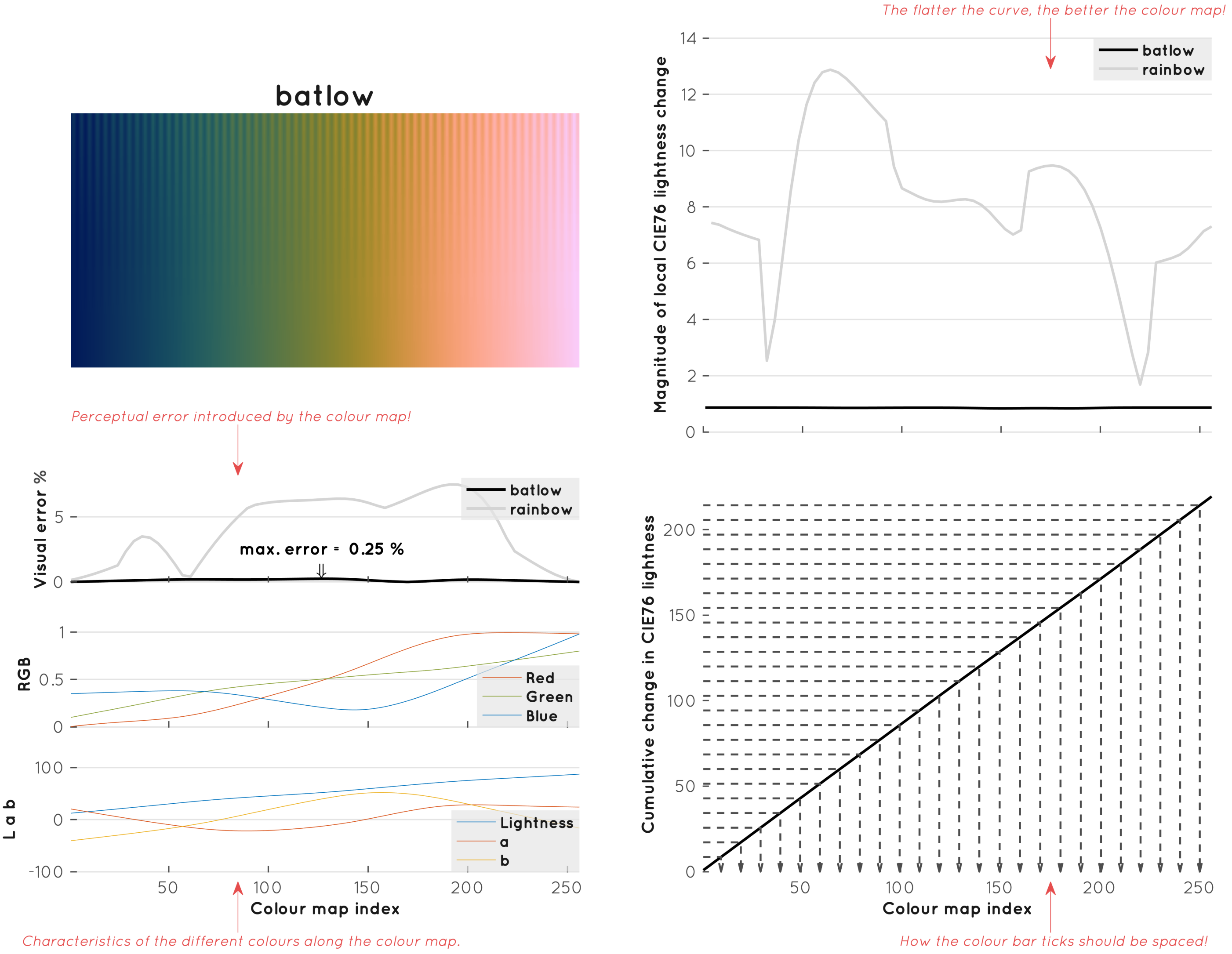

Supplementary Figure 5 Colour map diagnostics for batlow[1]**.** The figure shows the colour map with low gradient ripples to indicate how it represents low-contrast regions in different locations of the colour bar, and a plot of the lightness difference, ΔE, indicating better perceptual-uniformity the flatter the curve. Diagnostics are performed after Kovesi[3] and Crameri[4]. Similar diagnostics for all individual Scientific colour maps can be found on www.fabiocrameri.ch/colourmaps.

Supplementary Note 1: Better figures of widely used example datasets

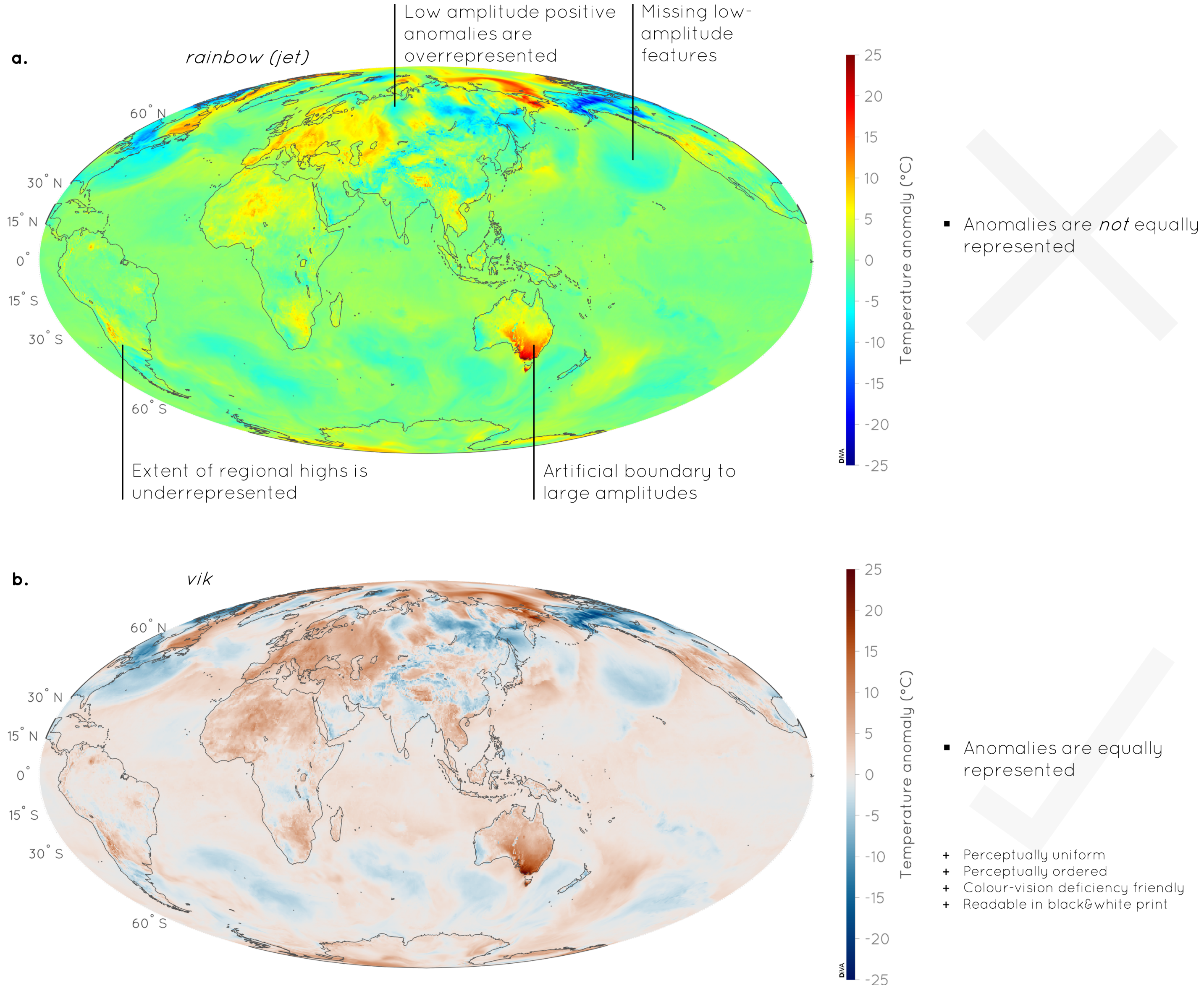

Supplementary Figure 6 Better data representation in weather and climate science. Global daily temperature anomaly represented with **a** the unscientific *jet* and **b** the scientific diverging *vik*[5] colour map. Shown is a NCEP GFS 0.5 degree analysis of daily 2m above ground air temperature anomaly of December 18, 2019, relative to the reference period 1981-2010 (Data kindly provided by Karsten Haustein; www.karstenhaustein.com/reanalysis/info.php). The Scientific colour map prevents alarmistic colouring, prevents visual data distortion, and makes the figure intuitively and universally readable.

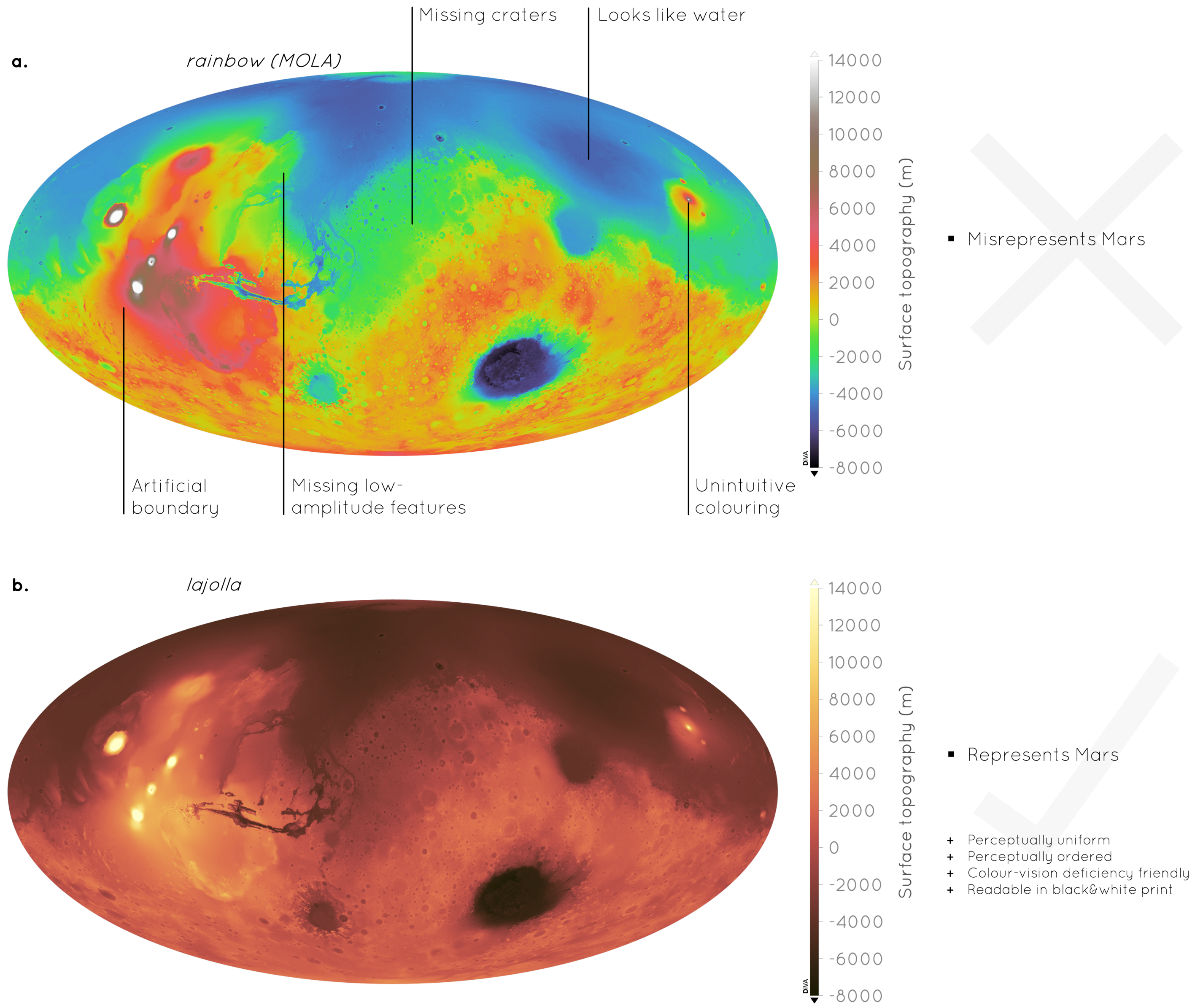

Supplementary Figure 7 Better data representation in planetary exploration. Martian surface topography represented with **a** the unscientific *MOLA rainbow* and **b** the scientific sequential *lajolla*[2] colour map. Shown is the digital elevation model (available from https://astrogeology.usgs.gov) based on Mars Orbiter Laser Altimeter data (MOLA[6]) obtained on NASA's Mars Global Surveyor (MGS) spacecraft[7]. The Scientific colour map prevents unnatural data representation, prevents visual data distortion, and makes the figure intuitively and universally readable.

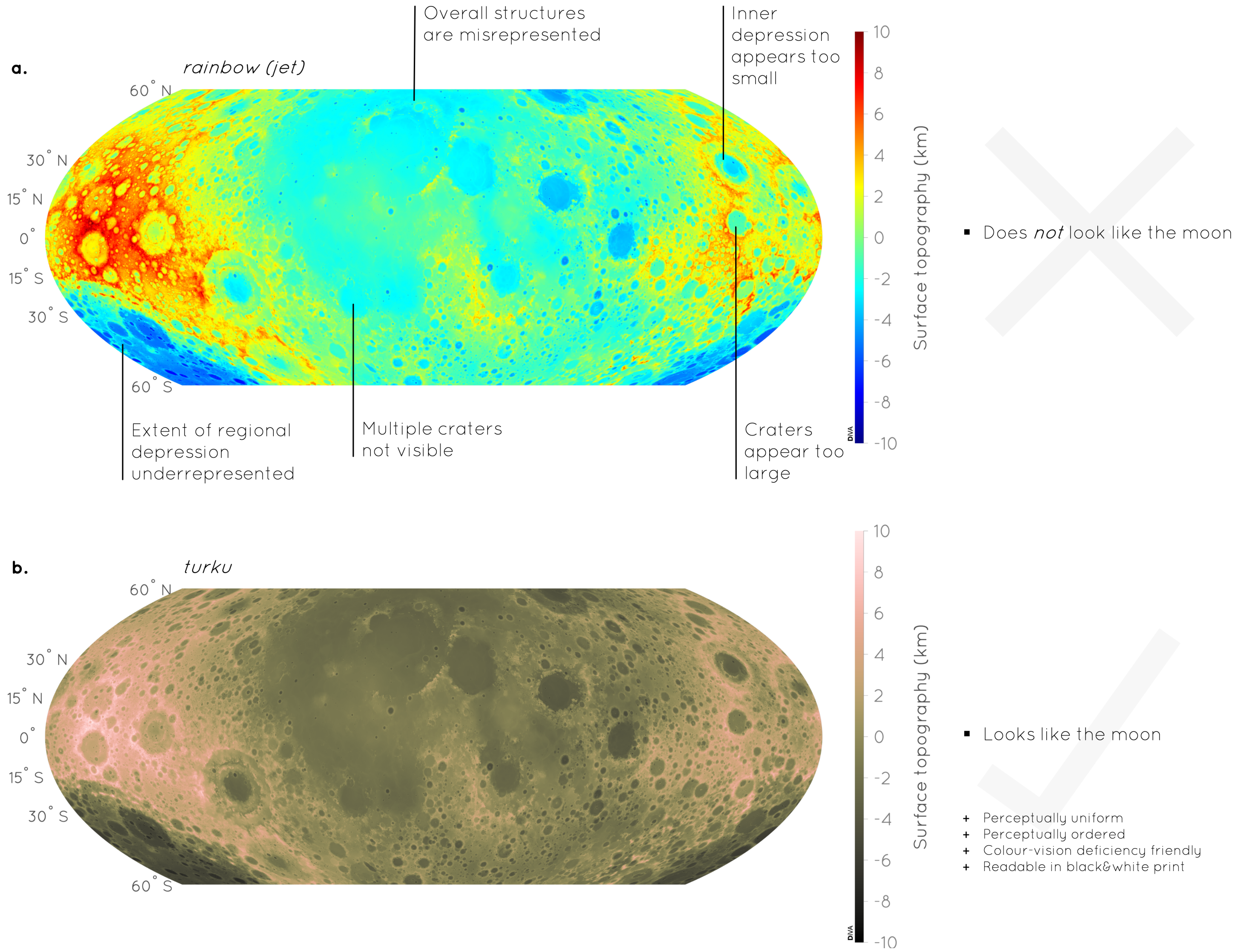

Supplementary Figure 8 Better data representation in planetary science. Lunar surface topography represented with **a** the unscientific *jet* and **b** the scientific sequential *turku*[8] colour map. Shown is DEMS data for ±60 degrees in latitude and 360 degrees in longitude, designated as SLDEM2015 and available from the Planetary Data System LOLA data node (http://imbrium.mit.edu/EXTRAS/SLDEM2015/). The Scientific colour map prevents unnatural data representation, prevents visual data distortion, and makes the figure intuitively and universally readable.

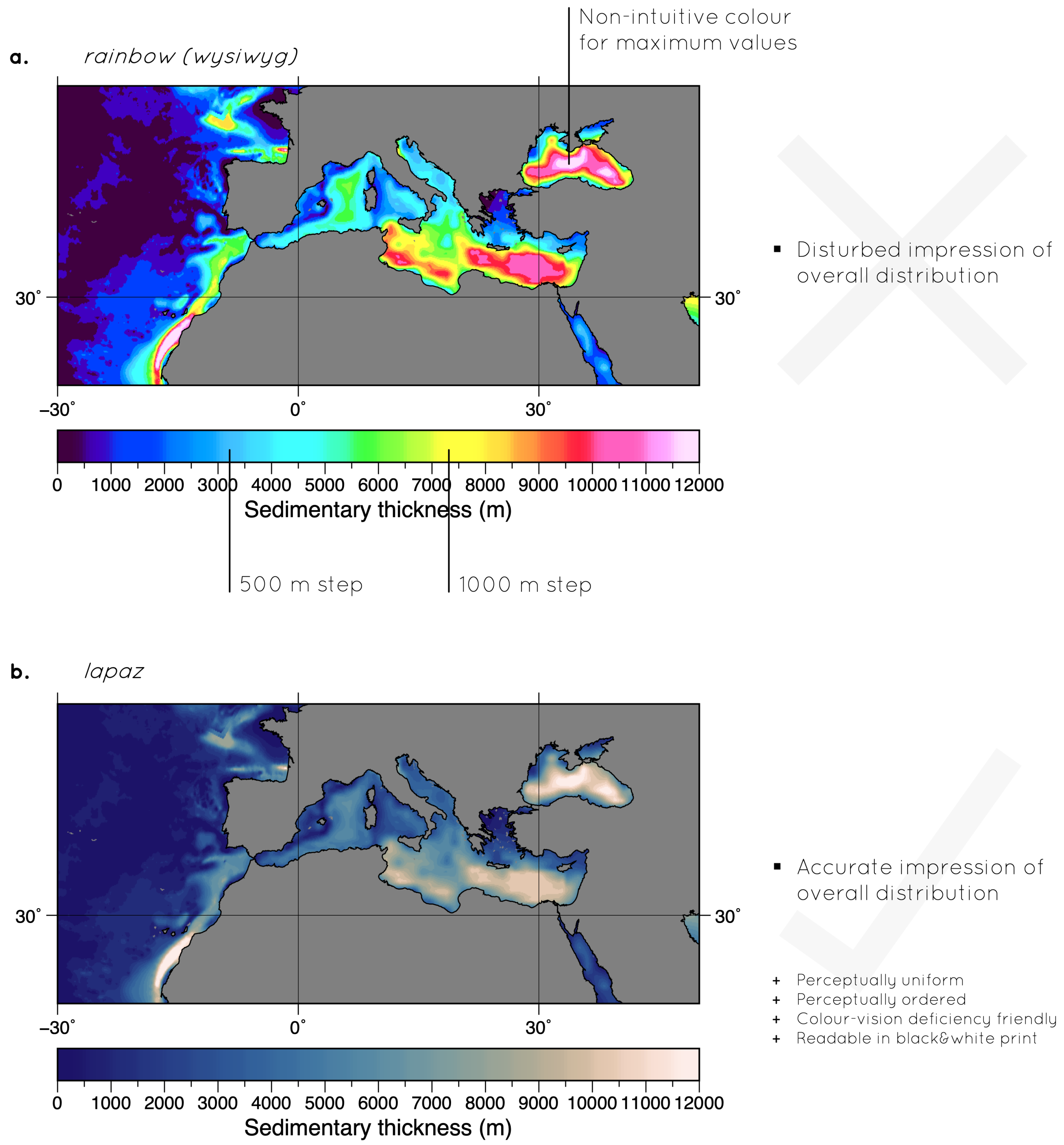

Supplementary Figure 9 Better data representation in ocean science. Ocean floor sediment cover presented with **a** the unscientific *wysiwyg* and **b** the scientific sequential *lapaz*[9] colour map. Shown is data from the GlobSed[10] global sedimentary thickness map in the Mediterranean region. The Scientific colour map prevents a disturbed impression of the overall sediment distribution, prevents other visual data distortion, and makes the figure intuitively and universally readable.

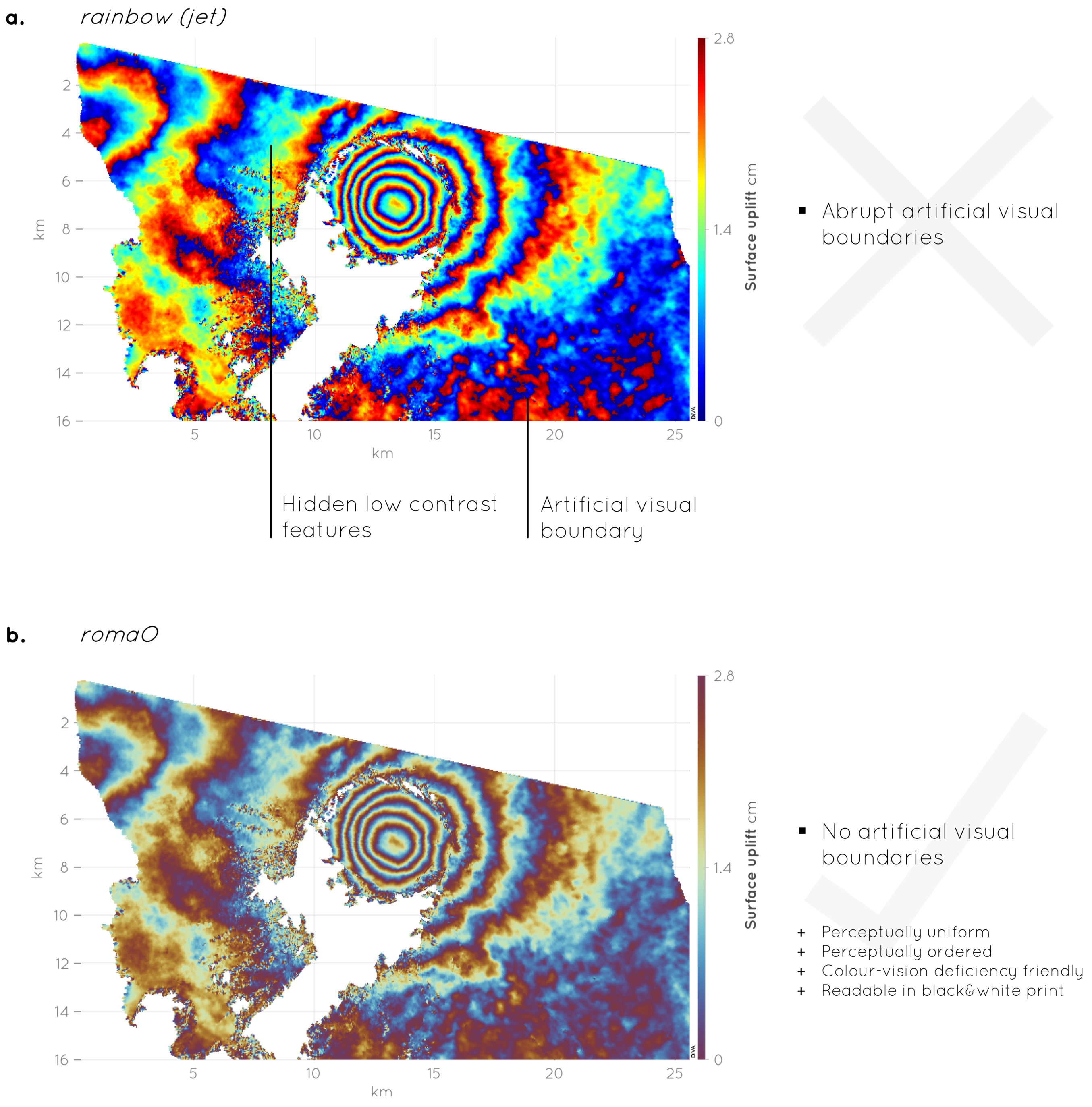

Supplementary Figure 10 Better data representation in Remote Sensing Science. The surface uplift over a given time period on the Darwin volcano (Galápagos Islands, Ecuador; example data set from Jónsson[11]) presented with **a** the unscientific *jet* and **b** the scientific cyclic *romaO*[12] colour map. The Scientific colour map prevents the sharp boundary between maximum and minimum values and other visual data distortion, and makes the figure intuitively and universally readable.

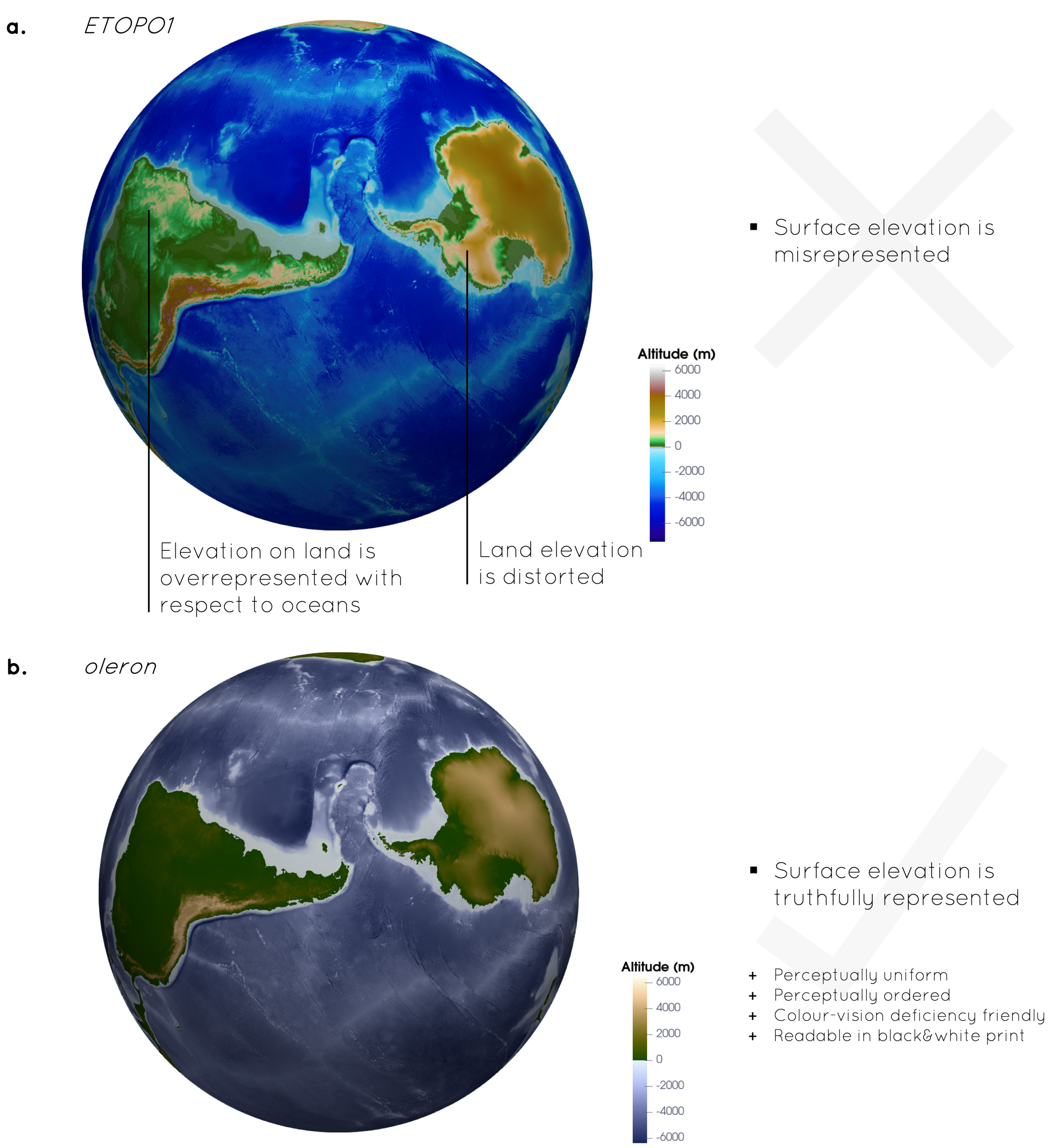

Supplementary Figure 11 Better data representation in Earth Surface Science. The surface surface topography on the Earth from the ETOPO1 dataset[13] presented with **a** the unscientific, standard *ETOPO1* and **b** the scientific multi-sequential *oleron*[14] colour map. The Scientific colour map prevents unfair representation of ocean and land elevation, prevents other visual data distortion, and makes the figure intuitively and universally readable.

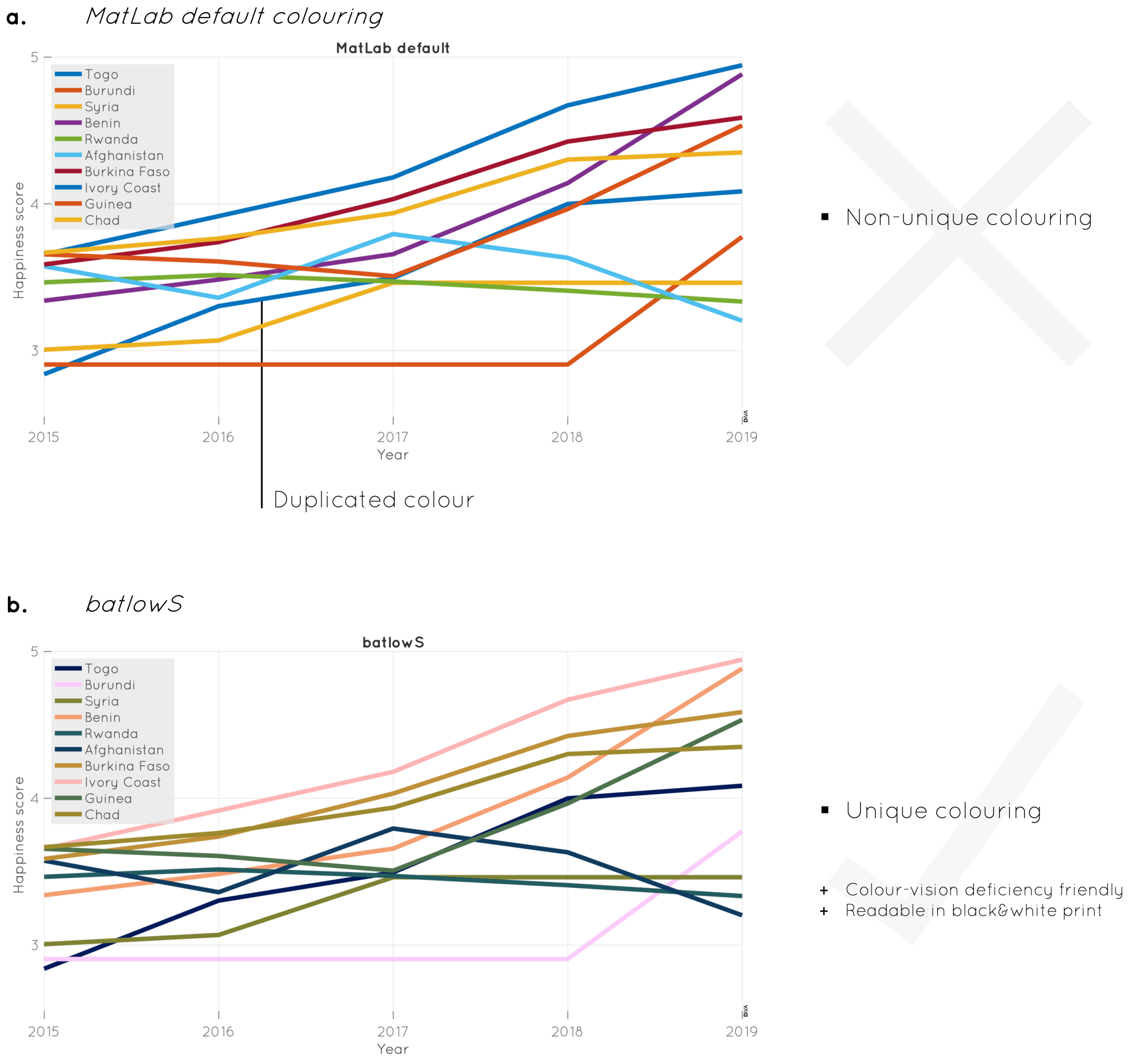

Supplementary Figure 12 Better data representation in Social Science. The World Happiness Report (`https://worldhappiness.report`) describes how measurements of well-being can be used effectively to assess the progress of nations. The openly available national happiness index (relative to Dystopia, a hypothetical country that has values equal to the world's lowest national average) are presented here with **a** the MatLab default colouring and **b** the scientific categorical *batlowS*[18] colour map. The Scientific colour map prevents repeating equal colours, while still ensuring clear discrimination between individual colours even for more than ten discrete data groups, while also being colour-vision deficiency and black&white friendly.

Supplementary Note 2: Colour map classes

Colour maps can be divided into four main classes with associated colour gradients, namely *sequential*, *diverging*, *multi-sequential*, and *cyclic*, that optimise the representation of four specific sets of data.

- **Sequential:** Sequential colour maps are optimal for sequential data with no particularly pivotal values or special characteristics. A given set of positive temperature values is, for example, best represented by a sequential colour map (see Supplementary Figures 5 and 6 for examples). Due to its general characteristics, sequential colour maps are the most versatile, can accurately represent most datasets, and are therefore the optimal default colour map in visualisation software. From the Scientific colour maps, the colour map *batlow*[1] is an example of a sequential class (see Figure 5). To highlight large values using the sequential class, the darker end of the colour map (e.g., the dark blue end of *batlow*) should represent those on light background. On dark background, it is the inverse and light colours (e.g., the light pink end of *batlow*) need to be used to make the high values appear most prominent (see also Supplementary Note 1).

- **Diverging:** Diverging colour maps place the focus on the centre point of the colour bar or, else, to the extreme values on either side[15]. As such, the diverging class is optimal to represent so-called bimodal datasets that contain data-points deviating from a central value. An example is a dataset of positive and negative temperature values deviating from the zero value (see Supplementary Figure 4 for an example). To use diverging colour maps properly, it is imperative to centre the middle of the colour bar and map to the central value of the dataset; the zero temperature value must, for example, lie exactly in the middle of the colour bar and map or else the data is misrepresented. To highlight the extreme values (for instance the most positive and negative temperatures), a colour map with dark edge values, like *roma*[16], has to be used on light background. On dark background, it is again the other way round and a colour map with light edge values and a dark centre (e.g., *berlin*[17]) has to be chosen (see also Supplementary Note 1).

- **Cyclic:** Cyclic (or orbital) colour maps are colour maps that have neither a beginning nor an end. They are useful to display periodic data sets like angular data or data from Synthetic Aperture Radar (SAR) interferometry, which is used to display Earth's surface displacement (see Supplementary Figure 7 for an example). The cyclic Scientific colour maps, like *romaO*[12], are diverging colour maps with matching ends. Since this class has no beginning or end, it is very versatile and does not have to be carefully adjusted to the underlying data.

- **Multi-sequential:** Multi-sequential colour maps are strongly data specific and as such are a special class that has to be carefully tuned to the underlying data. This particular colour map class consists of two or more sequential palettes arranged next to each other. As such they are similar to the *diverging* class, however, in the case of multi-sequential maps, the direction of lightness increase is the same for both segments. Like with diverging colour maps, the intermediate point, or points, have to be adjusted to represent some central value in the dataset. An example of a multi-sequential colour map is *oleron*[14], where the bluish part intends to represent surface topography below and the green-brownish part above the ocean; the point of the transition between the two sequential parts being fixed to the sea level (i.e., the zero topography value; see Supplementary Figure 8 for an example).

Supplementary Note 3: Colour map types

The constituent colours of a chosen colour palette can be further categorised into different types of colour maps which allow for their optimal use. A given palette of colours can either be arranged *continuously*, *discretely*, or *categorically* as is discussed in detail in the following sections.

- **Continuous:** Colour maps are generally displayed in a continuous type, using enough individual colour values to make, for example, a contour plot look smooth. Continuous colour maps are intended to display sets of continuous, ordered data points and can represent both small and large data variations. Continuous colour maps generally use more than a hundred individual colour values; the continuous Scientific colour maps (e.g., *batlow*[1]) use 256 colours (Figure 5). Due to the way perceptually uniform colour palettes are constructed, they naturally come in a continuous form, which is therefore available to the four different colour map classes (described in the previous section). A continuous colour map is also the most most versatile type, and the other types (discrete and categorical) can be constructed from it.

- **Discrete:** Discrete colour maps feature only a certain subset of all colour values of a pre-existing continuous colour map, that are clearly distinguishable from each other. Colour maps in discrete form are intended to visualise a set or range of discrete data points, but are also often used with continuous datasets. In the latter case, the author has to be careful to use enough discrete colours to not reduce the accuracy of the data presentation below values of interest; discretising the data set makes data points close to each other appear the same. Discrete colour maps are used with ordered data, and should be adjusted to the individual dataset to provide as many colours as the data is asking for. The discrete Scientific colour maps are provided with either 10 (as in Figure 5), 25, 50, or 100 individual colours. Any continuous colour map can be discretised. All the different colour map classes are therefore available in discrete form, too.

- **Categorical:** Categorical colour maps contain a high number of unordered unique colour values. They are intended to colour multiple individual data points or entire graphs to make them distinguishable from each other. Categorical colour maps can be applied to almost any type of graph, whether this is a scatter plot with multiple points, a line plot with multiple lines, a bar plot with multiple bars, or other types of graphs. The categorical type is therefore wonderfully versatile and widely used. Categorical Scientific colour maps are, however, almost non-existent. Individual palettes featuring the scientific attributes mentioned in the main manuscript are hardly available, and if they are, only contain very few (e.g., 5-10) colour values. In the age of big data, this is a major drawback. Here, we present for the first time scientifically derived categorical colour maps like, for example, *batlowS*[18] (Figure 5). They are based on the original, continuous Scientific colour maps[19] and therefore maintain all relevant scientific characteristics like CVD friendliness and grey-scale readability (see Supplementary Figure 9 for an example). We can provide categorical maps with a hundred individual colour values. Moreover, the individual colours have maximal colour and lightness contrast, which is largest for the first values and then decreases naturally with the total number of colours used. The largest possible contrast amongst all applied colours is always maintained and, therefore, the optimal visual separation between them is used. In addition, the algorithm used to create them further applies a random element to prevent the unwanted order to the resulting colouring, and prevents black and white colour values, which would make some data-points unreadable on white or black background. Categorical types are provided for all sequential Scientific colour maps. Diverging, multi-sequential, or cyclic colour maps cannot be used to create disordered categorical colour maps, since similar lightness values would make individual colour values indistinguishable in grey-scale and, possibly, for colour-deficient vision.

Supplementary Note 4: Colour spaces background

Colour spaces contain a large number of colours visible to the human eye and can be a useful tool to represent or extract certain colours or colour gradients. To represent colours, the red-green-blue (sRGB) colour space is used most commonly, and represents a certain colour by its red, green, and blue (RGB) content. RGB is not only commonly used to specify a certain colour, but also to reproduce and display it on digital displays. As convenient as the sRGB colour space is to describe and visualise individual colours, it only contains a limited portion of all colours visible to the human eye and is not useful to represent changes between them: there is no correlation between changes of RGB colour values and our perception of these changes. A colour space that represents our perceived changes in colour equally in numbers would be optimal. The first such colour space, the CIE 1931 XYZ colour space[20], was created by the International Commission on Illumination (CIE) and is still used today. Since the perception of a certain colour also depends on the viewing conditions (see Box 1), so-called colour appearance models (CAMs), and later even image appearance models (iCAMs)[21], have been used to produce even more accurate colour spaces.

Supplementary References

1. Crameri, F. The Scientific colour map 'batlow', 2020. URL http://www.fabiocrameri.ch/batlow.
2. Crameri, F. The Scientific colour map 'lajolla', 2020. URL http://www.fabiocrameri.ch/lajolla.
3. Kovesi, P. Good colour maps: How to design them. *CoRR*, abs/1509.03700, 2015. URL http://arxiv.org/abs/1509.03700.
4. Crameri, F. Geodynamic diagnostics, scientific visualisation and StagLab 3.0. *Geoscientific Model Development*, 11(6):2541–2562, 2018. doi: 10.5194/gmd-11-2541-2018. URL https://www.geosci-model-dev.net/11/2541/2018/.
5. Crameri, F. The Scientific colour map 'vik', 2020. URL http://www.fabiocrameri.ch/vik.
6. Smith, D. E., W. L. Sjogren, G. L. Tyler, G. Balmino, F. G. Lemoine, and A. S. Konopliv. The Gravity Field of Mars: Results from Mars Global Surveyor. *Science*, 286(5437):94, 10 1999. doi: 10.1126/science.286.5437.94. URL http://science.sciencemag.org/content/286/5437/94.abstract.
7. Albee, A. L., R. E. Arvidson, F. Palluconi, and T. Thorpe. Overview of the Mars Global Surveyor mission. *Journal of Geophysical Research: Planets*, 106(E10):23291–23316, 2020/06/20 2001. doi: 10.1029/2000JE001306. URL https://doi.org/10.1029/2000JE001306.
8. Crameri, F. The Scientific colour map 'turku', 2020. URL http://www.fabiocrameri.ch/turku.
9. Crameri, F. The Scientific colour map 'lapaz', 2020. URL http://www.fabiocrameri.ch/lapaz.
10. Straume, E. O., C. Gaina, S. Medvedev, K. Hochmuth, K. Gohl, J. M. Whittaker, R. Abdul Fattah, J. C. Doornenbal, and J. R. Hopper. GlobSed: Updated total sediment thickness in the world's oceans. *Geochemistry, Geophysics, Geosystems*, 20(4):1756–1772, 2019/12/11 2019. doi: 10.1029/2018GC008115. URL https://doi.org/10.1029/2018GC008115.
11. Jónsson, S. *Modeling volcano and earthquake deformation from satellite radar interferometric observations.* PhD thesis, Stanford University, Ann Arbor, 2002. URL https://search.proquest.com/docview/305523554?accountid=27229.
12. Crameri, F. The Scientific colour map 'romaO', 2020. URL http://www.fabiocrameri.ch/cycliccolourmaps.
13. Amante, C. and B. Eakins. ETOPO1 1 arc-minute global relief model: Procedures, data sources and analysis. NOAA Technical Memorandum NESDIS NGDC-24, National Geophysical Data Center, NOAA, Colorado, 2009.
14. Crameri, F. The Scientific colour map 'oleron', 2020. URL http://www.fabiocrameri.ch/oleron.
15. Moreland, K. Diverging color maps for scientific visualization. In Bebis, G., R. Boyle, B. Parvin, D. Koracin, Y. Kuno, J. Wang, R. Pajarola, P. Lindstrom, A. Hinkenjann, M. L. Encarnação, C. T. Silva, and D. Coming, editors, *Advances in Visual Computing*, pages 92–103, Berlin, Heidelberg, 2009. Springer Berlin Heidelberg. ISBN 978-3-642-10520-3.
16. Crameri, F. The Scientific colour map 'roma', 2020. URL http://www.fabiocrameri.ch/roma.
17. Crameri, F. The Scientific colour map 'berlin', 2020. URL http://www.fabiocrameri.ch/berlin.
18. Crameri, F. Categorical Scientific colour maps like 'batlowS', 2020. URL http://www.fabiocrameri.ch/categorical.
19. Crameri, F. Scientific colour maps. Zenodo, May 2018. URL https://www.doi.org/10.5281/zenodo.1243862.
20. Smith, T. and J. Guild. The CIE colorimetric standards and their use. *Transactions of the optical society*, 33(3):73, 1931.
21. Fairchild, M. D. and G. M. Johnson. iCAM framework for image appearance, differences, and quality. *Journal of Electronic Imaging*, 13 (1):126–139, 2004.
