# Supplementary material for: The misuse of colour in science communication
Source: Nat Commun. 2020 Oct 28;11:5444. doi: 10.1038/s41467-020-19160-7 (PMC7595127; doi:10.1038/s41467-020-19160-7)
Supplement: Supplementary file 2 — Description of Additional Supplementary Files [file 41467_2020_19160_MOESM2_ESM.pdf]

Description of Additional Supplementary Files

File Name: Supplementary Data 1
Description: Outreach poster to inform and remind about the importance of a scientific use of colours.

File Name: Supplementary Movie 1
Description: Colour and lightness perception. Equally coloured objects might misleadingly appear to have different hue or lightness even though they do not and indeed represent the exact same values (an effect also known as “checker shadow illusion”). An effect that becomes problematic for scientific figures, as for example with heatmap plots (see Supplementary Figure 3). Movie “Colour and lightness perception” is created and provided by Stefan Scherrer, at Zurich University of the Arts, 2018.
