# Supplementary material for: The misuse of colour in science communication
Source: Nat Commun. 2020 Oct 28;11:5444. doi: 10.1038/s41467-020-19160-7 (PMC7595127; doi:10.1038/s41467-020-19160-7)
Supplement: Supplementary file 3 — Supplementary Data 1 [file 41467_2020_19160_MOESM3_ESM.pdf]

Scientific colour maps

A "scientific colour map" uses a methodology that **prevents data distortion**, offers **intuitive colouring**, and is **accessible for people with colour-vision deficiencies**. However, most scientists use colour maps that distort data through uneven colour gradients (e.g., rainbow):

- ✓ Intuitive
- ✓ Distortion free
- ✓ Inclusive
- ✓ Freely available

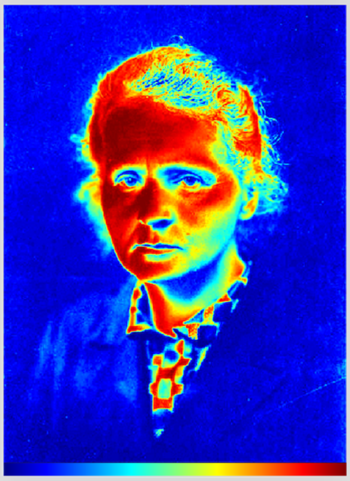

jet

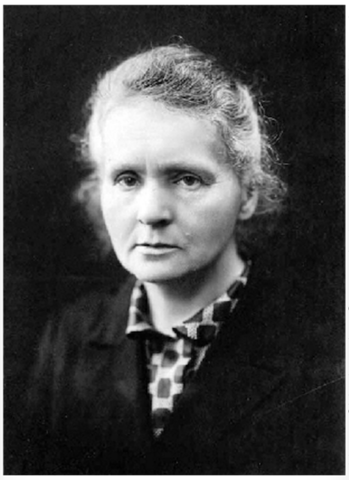

Marie Sklodowska-Curie, photographed by Henri Manuel

original

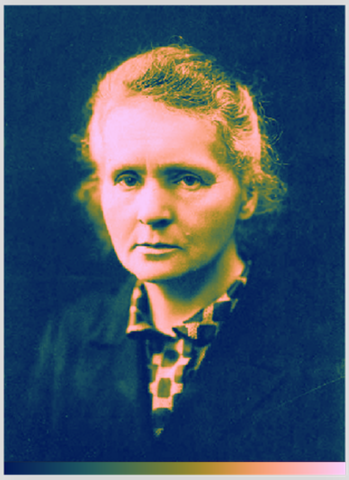

batlow

For every figure, think... is it scientific?

Your software... look beyond the default!

Your poster... accessible to the colour blind?

Your peers... say 'no' to rainbow!

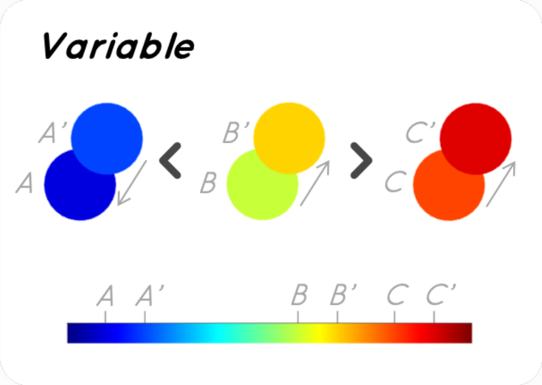

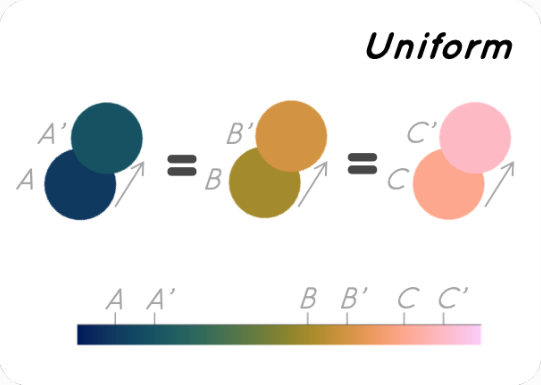

Incremental colour contrast

For maps and more

Download the paper

www.fabiocrameri.ch
